# CNS-Targeted Production of IL-17A Induces Glial Activation, Microvascular Pathology and Enhances the Neuroinflammatory Response to Systemic Endotoxemia

**DOI:** 10.1371/journal.pone.0057307

**Published:** 2013-02-27

**Authors:** Julian Zimmermann, Marius Krauthausen, Markus J. Hofer, Michael T. Heneka, Iain L. Campbell, Marcus Müller

**Affiliations:** 1 Department of Neurology, Universitätsklinikum Bonn, Bonn, Germany; 2 Department of Neuropathology, University Clinic of Marburg and Giessen, Marburg, Germany; 3 Clinical Neuroscience Unit, University of Bonn, Bonn, Germany; 4 School of Molecular Bioscience, University of Sydney, Sydney, Australia; Friedrich-Alexander University Erlangen, Germany

## Abstract

Interleukin-17A (IL-17A) is a key cytokine modulating the course of inflammatory diseases. Whereas effector functions of IL-17A like induction of antimicrobial peptides and leukocyte infiltration could clearly be demonstrated for peripheral organs, CNS specific effects are not well defined and appear controversial. To further clarify the functional significance of IL-17A in the CNS, we generated a transgenic mouse line with astrocyte-restricted expression of the IL-17A gene. GFAP/IL-17A transgenic mice develop normally and do not show any signs of neurological dysfunction. However, histological characterization revealed astrocytosis and activation of microglia. Demyelination, neurodegeneration or prominent tissue damage was not observed but a vascular pathology mimicking microangiopathic features was evident. Histological and flow cytometric analysis demonstrated the absence of parenchymal infiltration of immune cells into the CNS of GFAP/IL-17A transgenic mice. In GFAP/IL-17A mice, LPS-induced endotoxemia led to a more pronounced microglial activation with expansion of a distinct CD45^high^/CD11b^+^ population and increased induction of proinflammatory cytokines compared with controls. Our data argues against a direct role of IL-17A in mediating tissue damage during neuroinflammation. More likely IL-17A acts as a modulating factor in the network of induced cytokines. This novel mouse model will be a very useful tool to further characterize the role of IL-17A in neuroinflammatory disease models.

## Introduction

Recently, a number of studies point toward a central role for the interleukin-17 (IL-17) cytokine family in various CNS diseases [1]. The IL-17 cytokine family consists of six members named IL-17 (IL-17A), IL-17B, IL-17C, IL-17D, IL-17E (IL-25) and IL-17F [2]. The most prominent members are IL-17A and IL-17F which form functional homo- or hetero-dimers with largely overlapping proinflammatory effects bridging the adaptive and innate immune response [3]-[5]. Effector functions of IL-17A are considered pivotal in the host response against extracellular and intracellular pathogens [6]-[8] and are associated with the pathogenesis of many autoimmune inflammatory diseases [9]-[14]. There is a convincing body of evidence that IL-17A plays an important role in inflammatory brain disorders including multiple sclerosis [15], infectious CNS diseases [16] and stroke [17], [18] as well as in the pathophysiology of vascular inflammation and arteriosclerosis [19], [20]. In these pathological conditions, the source of IL-17A can vary from infiltrating hematogenous immune cells like Th17 polarized CD4^+^ T-cells [21], [22], CD8^+^ T-cells, gammadelta T-cells [23], NK-cells [24], and granulocytes [25], [26] to CNS resident cells. In particular astrocytes have been demonstrated to secrete IL-17 in pathological conditions like multiple sclerosis and ischemic brain injury [15], [17,].

Th17 polarized T-cells came into focus of research after the pivotal role of IL-23 in the induction of EAE was described almost a decade ago [27] (reviewed in [28]). This finding resolved contradicting results that challenged the concept that organ specific autoimmunity was a Th1 driven condition: mice genetically-deficient in IFN-γ and IFN-γ receptor, as well as mice with impaired Th1 differentiation were not protected from EAE but developed more severe disease [29], [30]. IL-23 induces the proliferation of a IL-17 secreting independent T-cell subset subsequently named Th17 cells [10], [31], [32]. To induce Th17 lineage commitment, stimulation of naïve T-cells with a combination of TGF-β and IL-6 [33]–[35] or with a combination of IL-21 and TGF-β [36] is required.

The receptor for IL-17A and IL-17F consists of a heterodimeric complex of IL-17RA and IL-17RC and is expressed in the CNS on astrocytes, microglia and endothelial cells [37], [38]. Its stimulation induces NFkappaB and MAP kinase activation via TRAF6 and the adaptor protein Act-1 signaling [39], [40] thus leading to the expression of many proinflammatory cytokines, chemokines and antimicrobial peptides. Particularly IL-17A is involved in the expansion and recruitment of neutrophils through the induction of G-CSF and the ELR+ members of the CXC family of chemokines CXCL1 and CXCL2 [41]–[43]. However, though effector functions of IL-17A are well characterized outside the brain, the direct CNS effector functions remain vague. *In vitro* data suggests an activation of microglia and synergistic effects of IL-6 stimulation on astrocytes through IL-17A signaling [44], [45]. Furthermore, IL-17A is thought to disrupt the blood brain barrier by release of reactive oxygen species [39], [46]. *In vivo,* there are few and partly controversial data regarding the impact of IL-17A on CNS autoimmune diseases. Whereas in EAE, genetic deletion or neutralization of this cytokine resulted in an attenuated disease course in some studies [47]–[50], it was shown recently in other studies that mice lacking IL-17A and IL-17F were still susceptible to EAE [51]. Disruption of IL-17A signaling pathways by genetic knockout of the IL-17 receptor subunit IL-17RC [52] or astrocyte targeted deletion of Act1 is highly capable of ameliorating EAE disease course [53].

Furthermore, knowledge about the impact of IL-17A on CNS infections is limited and comparably contradictory [16], [54], [55].

As outlined above, it is clear that IL-17A is a multifunctional cytokine with direct effects on CNS resident and infiltrating cells. However, at present most data addressing CNS effector functions of IL-17A are from *in vitro* experiments and we lack appropriate models for dissecting the functional properties *in vivo*. To examine the functional impact of IL-17 on the CNS *in vivo*, we have generated a transgenic mouse with astrocyte-specific production of IL-17A under the transcriptional control of a well-described glial fibrillary acidic protein (GFAP) genomic expression vector [56]-[58].

## Materials and Methods

### Animals

The method used for the generation of transgenic mice with astrocyte-targeted gene expression under the transcriptional control of a GFAP promoter was described in detail previously [57]. In brief, the coding sequence for the murine *Il17a* gene (bases 58-534; GenBank accession no. NM_010552) was amplified by RT-PCR from RNA isolated from the spleen of mice suffering from EAE at peak clinical disease. The oligonucleotide primers aagtgcacccagcaccag (5’) and cgcgggtctctgtttagg (3’) were used for the PCR. After digestion with the appropriate restriction enzymes and subsequent sequence verification, the amplified *Il17a* cDNA fragment was cloned into a GFAP expression vector containing a human growth hormone polyadenylation signal sequence downstream of the insert as described previously [58]–[59]. The resulting fusion gene construct was microinjected into the germline of (C57Bl/6×C3H/HeN) F1 mice. Genotyping of the animals was accomplished by PCR analysis of genomic tail DNA using primers targeted at the human growth hormone sequence and the *Il17a* sequence included in the transgene construct. Hemizygote transgenic founder mice were backcrossed to the C57BL/6 background for at least 8 generations before experiments were performed. Transgene negative mice served as wild-type littermate controls. All mice were maintained under pathogen-free conditions in the closed breeding colony of the University Hospital of Muenster, Germany. All animal experiments were approved by the Animal Care Commission of Nordrhein-Westfalen.

### Tissue processing for histology

The tissue for analysis was obtained from GF/IL17 hemizygote animals and littermate controls at different ages as indicated in the results and figures. Brains were removed for routine histological and immunohistochemical examination. Immediately after euthanasia, brains were removed and brain halves (cut along the sagittal midline) were fixed overnight in PBS-buffered 4% paraformaldehyde at 4 °C, washed in PBS and subsequently embedded in paraffin. Sections (5 µm) were prepared from paraffin-embedded tissue. For immunohistochemistry on cryosections, tissue was embedded with Tissue Tek (Sakura Finetek, Staufen, Germany) after cryoprotection in 30 % (w/v) sucrose. For fluorescence microscopy 8 µm sections, for confocal laser scanning microscopy 50 µm sections were prepared. To ensure the same staining conditions sections from transgenic GF/IL17 animals were always mounted together with sections from control mice on a single glass slide.

### Primary astrocytes cell culture

Primary mixed glial cell cultures were prepared from single 1 d old hemizygote GF/IL17 and wildtype littermate mice as previously described with modifications [60]. Briefly, mixed glial cultures were prepared from newborn mice and cultured in DMEM supplemented with 10% FCS and 100 U/ml penicillin/streptomycin. After 10–14 d of primary cultivation floating microglial cells were discarded after intensive shaking. Remaining astrocytes were extensively washed with culture medium and kept in culture for 12 hours. Supernatants were harvested and snap frozen in liquid nitrogen, whereas astrocytes were dislodged by mild trypsinization and pelleted for RNA extraction using RNAeasy Columns (Qiagen, Hilden, Germany) according to the Manufacturer’s instructions.

### Routine histology and immunohistochemistry

Paraffin-embedded sections were stained with H&E and Luxol fast blue for routine histological analysis and evaluation of myelination. For the detection of calcium deposits Alizarin red S staining (Sigma) was performed [61]. For immunohistochemistry of paraffin embedded tissue, sections were rehydrated in graded ethanol series after deparaffination in xylene. Slides were then incubated over night at 4°C with primary antibodies (primary antibodies and corresponding protocols for immunohistochemistry are summarized in Table 1). After washing in PBS, a corresponding biotinylated secondary antibody (Axxora, Lörrach, Germany; 1/200) and HRP-coupled streptavidin (Axxora; 1/200) was used. Nova Red (Vector Labs) was applied as the immunoperoxidase substrate according to the manufacturer’s instructions. Sections were counterstained with haematoxylin (Sigma-Aldrich). Fluorescent immunohistochemistry was applied to frozen sections. Primary antibodies were incubated over night at 4° C. After washing in PBS, an A594 or A488 fluorescence-conjugated secondary antibody (Invitrogen, Darmstadt, Germany; 1/200, 60 minutes) was used to visualize the primary antibody. Sections were counterstained with DAPI (Sigma-Aldrich, Munich, Germany). Conventional and immunofluorescence-stained sections were examined under a Nikon eclipse 800 bright-field and fluorescence microscope (Nikon, Düsseldorf, Germany). Bright field images and monochrome fluorescent images were acquired using a Spot flex camera and SPOT advanced 4.5 software (Diagnostic instruments, Sterling, MI).

**Table 1:** Antibodies/Lectin used for histology.

Antibody/Lectin (source)	Specificity	Dilution	
		Paraffin	Cryostat
Polyclonal rabbit anti-Iba1 reactive with human, mouse and rat Iba1 (Wako Chemicals, Neuss, Germany)	Microglia/ macrophages	–	1/500
Monoclonal rat anti- mouse CD68 (Serotec, Düsseldorf, Germany)	Microglia/ macrophages	–	1/200
Polyclonal rabbit anti-Laminin reactive with human and mouse Laminin (Sigma-Aldrich, Munich, Germany)	Basal lamina	–	1/50
Polyclonal rabbit anti -human GFAP (Dako, Hamburg, Germany)	Glial fibrillary acidic protein	1/200	–
Biotin-conjugated tomato lectin, *L. esculentum* (Axxora, Lörrach, Germany )	Microglia/ macrophages, endothelial cells	1/50	–
Monoclonal mouse anti-mouse NeuN (Chemicon, Schwalbach, Germany)	Neurons	1/200	–
Monoclonal mouse anti-mouse PLP (Serotec, Düsseldorf, Germany)	Proteo-Lipid-protein	1/500	–

### Analysis of vascular density by confocal laser microscopy

For confocal analysis of vascular density free-floating sagittal sections (50 µm) were permeabilized by 1% Triton X-100 for 2 hours at room temperature. Consecutive antibody incubation steps were performed in 1% Triton X-100 overnight at 4°C. Microvasculature was stained using rabbit anti-laminin antibody with a subsequent incubation with an A594-conjugated secondary antibody. Sections from mutant and wild-type animals were processed in parallel. 20 µm z-stacks were captured from corresponding regions (corpus callosum, hippocampus, white matter) using a Zeiss LSM510 laser scanning microscope. Capillaries were identified by a lumen diameter < 10 µm and microvascular network was analyzed using ImageJ software.

### Electron microscopy and analysis of basement membrane thickness

Brains were dissected after fixation in 3% glutaraldehyde. Corpus callosum dissections were later progressively dehydrated in a graded series of ethanol (30–100%) and embedded in Epon. Ultrathin sections (50–60 nm) were cut using an Ultracut microtome and placed on copper grids for analysis. Grids were stained and contrasted with uranyl acetate and lead citrate, followed by examination with Zeiss TEM900 electron microscope as described in detail before [62]. Basement membrane thickness was analyzed using ImageJ. Thickness was measured from saggital sectioned capillaries at three different positions where duplications of basement membrane were absent and mean values were calculated.

### Cytokine and Chemokine mRNA determination by qRT-PCR

RNA of whole brain hemispheres, dissected forebrains and cerebelli or spinal cord was isolated using Trizol reagent (Invitrogen) according to the manufacturer’s instructions. Total RNA (2 µg) was reverse-transcribed into cDNA using SuperScript™ III Reverse Transcriptase (Invitrogen). Real-time quantitative PCR assays were performed using Taqman reagents (Applied Biosystens, Darmstadt, Germany). Samples were analyzed simultaneously for *Gapdh* mRNA as the internal control. The mRNA levels for each target were normalized to mRNA levels of *Gapdh* and expressed relative to that of nontransgenic littermate controls. Each sample was assayed in duplicates. Predesigned Taqman assays (Applied Biosystems) were used to amplify the following targets: *Gapdh, Il17a, Tnf, Il1b, Il6, Cxcl1, Cxcl2, Ccl2, Cxcl10*, *Csf2* and *Mmp9*. cDNA samples from neuroinflammatory disease models (EAE, murine West Nile virus encephalitis (WNV), and experimental cerebral malaria (ECM)) were used to compare the CNS production of *Il17a* in the GFAP/IL17A transgenic mouse model with non-transgenic disease models. EAE and ECM were induced as described in detail before [63].

### CNS leukocyte isolation and flow cytometry

CNS microglia and parenchymal infiltrating leukocytes were isolated from whole brain homogenates as described previously [64] with modifications. In brief, mice were perfused transcardially with ice cold PBS until flow through was completely clear to remove intravascular leukocytes. After dissection brains were grinded in Hank’s Balanced Salt Solution (HBSS, Gibco, Eggenstein) using a tissue homogenizer (glass Potter, Braun, Melsungen) followed by a needle (0.6×25) and a syringe (5 ml) before passing through a 70 µm cell strainer (BD biosciences, Heidelberg). After pelleting, homogenates were resuspended in 75 % isotonic Percoll (GE-healthcare, Uppsala, Sweden) at 4°C. A discontinuous Percoll density gradient was layered as follows: 75 %, 25 % and 0% isotonic Percoll. The gradient was centrifuged for 25 min, 800 g at 4°C. Microglia, leukocytes, and astrocytes were collected from the 25 % / 75 % interface.

For surface marker staining the collected cells were directly washed in PBS, and blocked with CD16/CD32 (Fc block; eBioscience, Frankfurt/Main, Germany) antibody. Isolated leukocytes were incubated with fluorochrome-conjugated antibodies (eBioscience) to detect CD3e (PerCP-Cy5.5), CD11b (APC), CD11c (PE-Cy7), CD45 (FITC), CD45 (eFluor 450), Ly6G (PerCP-Cy5.5), B220 (APC-eFluor 780) and NK1.1 (PE-Cy7).

For intracellular cytokine staining cells were seeded in a 12 well plate at a density of 1×10^6^ cells / ml in DMEM (Gibco, Eggenstein) containing glutamine and 10 % FCS. Cells were incubated for 4 hours in the presence of LPS (100 ng/ml, Sigma) and Brefeldin A to block cytokine secretion according to manufacturer’s instructions (GolgiPlug, BD Biosciences). After blocking of Fc-receptors cells were surface stained with anti-CD45 (APC-Cy5.5) and anti-GLAST (APC, Miltenyi-Biotech, Bergisch Gladbach, Germany). Subsequently cells were washed, permeabilized using the Cytofix/Cytoperm kit (BD Biosciences) according to instructions, blocked again with Fc-block, and stained intracellulary with anti-TNFα (FITC, BD Biosciences).

After washing, bound Ab was detected using a BD FACSCanto II (BD Biosciences), and the acquired data were analyzed using the flow cytometry software, FlowJo (TreeStar, San Carlos, CA).

### Protein Lysates and Western Blot

Tissue was homogenized using a Precellys 24 tissue homogenizer *(*Bertin Technologies, Saint-Quentin-en-Yvelines Cedex, France*)* in lysis buffer described elsewhere [65]. Samples were centrifuged at 12000 rpm / 13200 g for 15 min and supernatants were taken. The protein concentrations were determined using the BCA Protein Assay Kit (Pierce, Rockford, IL). Protein lysates (50 µg) were separated by 10 % SDS-PAGE gel using NuPage MES SDS running buffer (Invitrogen) at 150 V. PageRuler Prestained Protein Ladder (Fermentas, St. Leon-Rot, Germany) was used as standard. Proteins were transferred to 0.2 µm nitrocellulose membranes (Whatman, Dassel, Germany). Membranes were blocked for 30 min in TBST containing 5% skim milk. Immunoblotting were performed using monoclonal anti-GFAP (Chemicon, Schwalbach, Germany) and antibody CP06 (Oncogene Science, Cambridge, MA) detecting α-tubulin followed by incubation with the appropriate horseradish-peroxidase conjugated secondary antibodies (Jackson ImmunoResearch, Newmarket, UK). Immunoreactivity was detected by chemiluminescence reaction (Millipore, Schwalbach, Germany) and luminescence intensities were analyzed using Chemidoc XRS imaging system (BioRad, Munich, Germany). With the Quantity One (BioRad) program bands density were determined for each lane and the intensity ratio for the detected GFAP were calculated to α-tubulin.

### Determination of serum and cell culture IL-17A protein level

Serum IL-17A was determined by ELISA (eBioscience) after cardiac puncture and centrifugation from GF/IL17 mice and littermate controls according to the manufacturer’s protocoll. For the in vitro determination of IL-17A-secretion, primary astrocyte cultures were incubated in fresh DMEM containing 10 % FCS for 12 hours and supernatant was harvested. A standard curve was generated according to the Manufacturer’s protocol.

### Induction of endotoxemia

Endotoxemia was induced by a double intraperitoneal injection of 100 µg LPS (Escherichia coli 026:B6; Sigma, Munich, Germany) 24 hours and 4 hours before killing in 5 GF/IL17 transgenic mice and 5 WT littermate controls. Mice were perfused with ice-cold PBS and subsequently brains were dissected in the sagittal midline immediately. One half was immediately snap-frozen in liquid nitrogen and stored at -80°C until RNA isolation. The other half was homogenised for FACS-analysis as described above.

### Analysis of blood brain barrier (BBB) integrity with Evans blue dye (EBD)

BBB integrity was determined with EBD as described previously [66], [67] with modifications. 3 hours before scarification 3 mice per group received intraperitoneal injections of EBD (2 % w/v in isotonic NaCl, 4 mg / kg body weight, AppliChem, Darmstadt, Germany). To remove intravascular EBD before removal of CNS tissue mice were perfused with 60 ml of ice-cold PBS supplemented with 2 mM EDTA (Sigma). The effective clearance of dye by the perfusion step was confirmed by the appearance of a colorless perfusate. Brain was dissected into forebrain and cerebellum, spinal cord and portions of liver were removed, washed briefly with double distilled H2O and weighed. Tissue from LPS treated mice served as positive control. Tissue was homogenized in a three-fold volume of 50% trichloroacetic acid (w/v, AppliChem) solution and pelleted. Supernatants were diluted with ethanol (1:3), and fluorescence was quantified using a microplate fluorescence reader (Tecan infinite 200 M, Crailsheim, Germany), (excitation: 620 nm, emission: 680 nm). Sample value calculations were based on Evans blue dye standards mixed with the same solvent.

### Statistical analysis

For statistical analysis, GraphPad Prism was used. Real time PCR data, ELISA, Western blot data, Evans blue dye extravasation data, or flow cytometry data were analyzed where appropriate by a two-tailed Student's t test with p<0.05 considered to be statistically significant.

## Results

### Generation of GFAP-IL-17 transgenic mice and analysis of transgene expression: chronic CNS IL-17A stimulation neither induces major tissue damage, neurodegeneration nor demyelination

To characterize the effects of IL-17A in the CNS we used a well-established approach, targeting the expression of cytokine transgenes into astrocytes [68]. We detected 18 transgene positive founder mice and generated two independent mouse lines (GF/IL17–15 and GF/IL17–45) with stable genomic integration of the GFAP-IL-17A construct. A comparison of peripheral organs from GF/IL17 and wild-type mice revealed no significant differences of *Il17a* mRNA between the two groups (liver, spleen, kidney, gut, lung, heart, hamstring muscle, and sciatic nerve). IL-17A protein was not detectable in serum of both transgenic mice and littermate controls, respectively (data not shown). Hemizygous transgenic animals up to the age of 18 months developed normally without showing clinical signs of neurological disease or obvious behavioral abnormalities. In addition, none of the founder mice showed a clinical or histopathological phenotype (data not shown).

**Figure 1:**
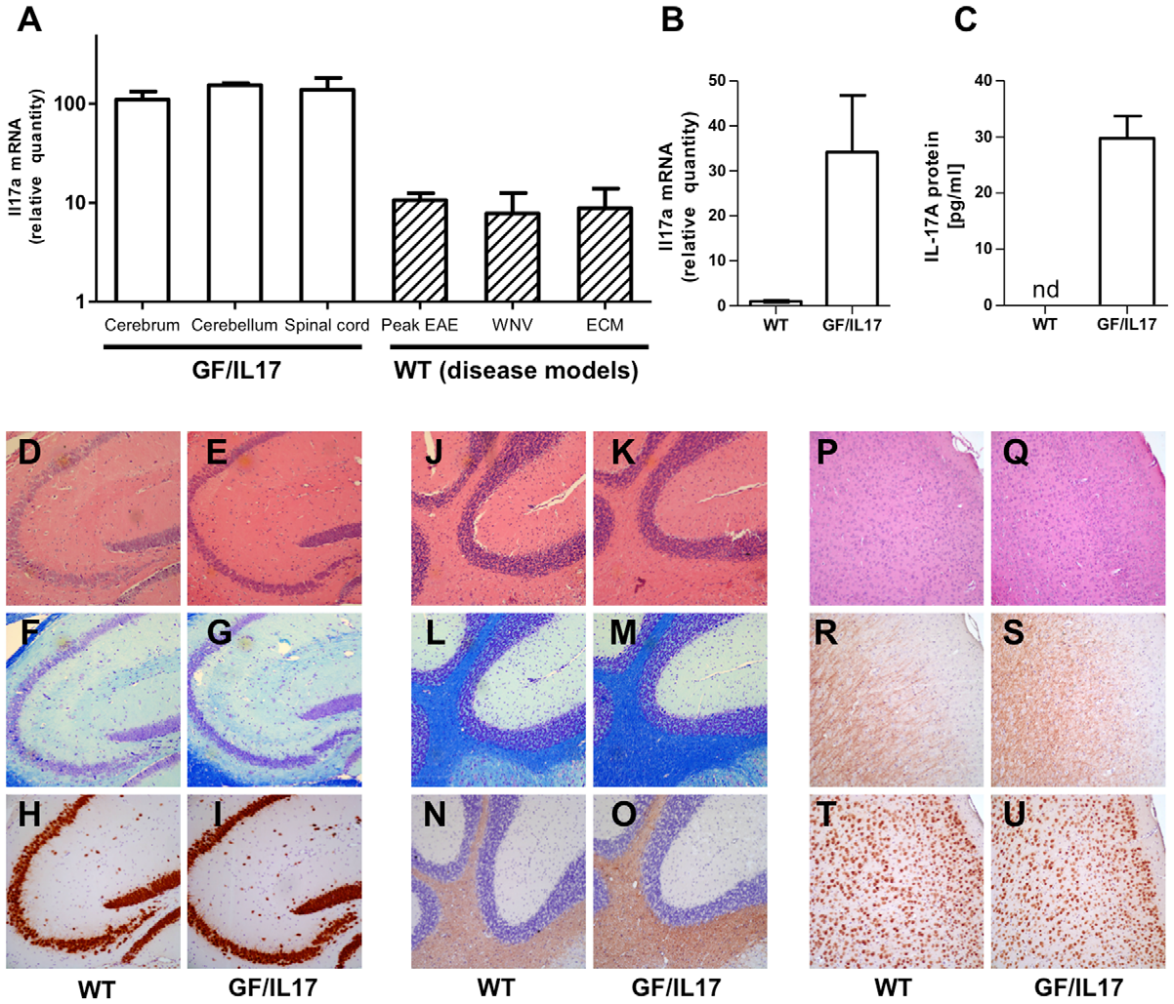
GF/IL17 mice express IL-17A mRNA and protein without major histological defects or leukocyte infiltration. **(A)** Relative expression of *Il17a* mRNA in GF/IL17 mice is equally distributed between forebrain, hindbrain and spinal cord. In comparison to disease models in WT mice (peak EAE, West Nile Virus encephalitis, experimental cerebral Malaria) CNS expression levels of *Il17a* in otherwise untreated GF/IL17 mice exceed levels of all tested disease models irrespective of the cellular source. **(B)** *Il17a* mRNA expression from GF/IL17 primary astrocytes and WT controls was quantified using real time PCR. **(C)** Astrocyte secretion of IL-17A protein was confirmed using ELISA. Supernatants of GF/IL17 and WT control primary astrocytes were analyzed after 12 hours of culture. In supernatant of WT control astrocytes IL-17A protein was not detectable. **(D**–**U)** Routine histological characterization of mice excluded major tissue damage or leukocyte infiltration. Representative areas of hippocampus **(D**–**I)** , cerebellum **(J**–**O)**, or cortex **(P**–**U)** are shown in WT controls **(D, F, H, J, L, N, P, R, T)** and GF/IL17 transgenic mice **(E, G, I, K, M, O, Q, S, U)** by HE **(D, E, J, K, P, Q)**, LFB **(F, G, L, M)**, anti murine NeuN mAb **(H, I, T, U)**, or anti murine PLP mAb **(N, O, R, S)** staining (age 9 month).

Analysis of relative transgene expression by quantitative PCR compared to WT whole brain lysates exhibited a comparable distribution of transgene encoded *Il17a* mRNA in forebrain (110.1 ± 22.6), cerebellum (153.9 ± 7.6), and spinal cord (137.9 ± 44.2) for both transgenic mouse lines (Fig. 1A). We further examined *Il17a* mRNA induction in viral and autoimmune inflammatory disease models to relate their *Il17a* mRNA induction with the level in our transgenic model. Compared with peak EAE, WNV encephalitis and ECM in C57Bl/6 WT mice CNS *Il17a* expression was significantly higher in the GF/IL17A mice (peak EAE: 10.6 ± 1.8, WNV 7.8 ± 4.6, ECM: 8.8 ± 5.1, for all p < 0.0001 compared to GF/IL17 mice). To confirm astrocyte IL-17A transcription and translation into protein with subsequent secretion we generated primary astrocyte cultures from GF/IL17–15 hemizygote mice and littermate controls. The presence of *Il17a* mRNA was detected using real time PCR (Fig. 1B) along with the confirmation of IL-17A protein secretion into the supernatant of GF/IL17 primary astrocyte cultures using ELISA (Fig. 1C). The further studies described below were conducted using the GF/IL17–15 line.

Routine histologic analysis of H&E-stained paraffin embedded sections was applied to detect histopathological alterations or cellular infiltrations in the CNS of GF/IL17 mice (Fig. 1E, 1K, 1Q) at different ages (1 – 12 month) compared with age- and sex- matched wild-type littermate controls (Fig. 1D, 1J, 1P). Chronic IL-17A production did not induce demyelination in GF/IL17 mice shown by luxol fast blue staining (Fig. 1F, 1G, 1L, 1M) or anti-PLP immunohistochemistry (Fig. 1N, 1O, 1R, 1S). Numbers and distribution of neurons were normal in GF/IL17 mice compared with wildtype controls using anti-NeuN immunohistochemistry (Fig. 1H, 1I, 1T, 1U). Taken together these findings indicate that the chronic astrocyte production of IL-17A in GF/IL17 transgenic mice is neither associated with a clinical phenotype nor with overt histological alterations.

### CNS specific IL-17A production neither alters the expression levels of inflammation-related genes nor promotes leukocyte infiltration into the CNS

We next determined whether the expression of a variety of IL-17A inflammation-related genes might be altered in the brains of GF/IL17 mice. With the exception of the transgene-encoded IL-17A, cerebral expression of the proinflammatory cytokines *Tnf, Il1b, Il6, Csf2,* the chemokines *Cxcl1, Cxcl2, Ccl2,* and the matrix degrading enzyme *Mmp9* were not induced in GF/IL17 mice compared with matched wild-type littermates (Table 2).

**Table 2:** Regulation of inflammation related genes in GF/IL17 mice relative to littermate controls (arbitrary units).

Gene	*Il17a*	*Tnfa*	*Il1b*	*Il6*	*Csf2*	*Cxcl1*	*Cxcl2*	*Ccl2*	*Mmp9*
Rel. expression	111 ± 11***	1.0 ± 0.07	1.2 ± 0.11	0.9 ± 0.05	0.9 ± 0.05	1.0 ± 0.04	1.1 ± 0.06	0.9 ± 0.05	1.0 ± 0.03

To further exclude leukocytic infiltrates in the brain of GF/IL17 mice, flow cytometric analysis was performed (Table 3). No differences were found in the cellular ratios of CD45^dim^/CD11b^+^ microglia, CD45^+^/Ly6G^+^ granulocytes, CD45^+^/CD3^+^ T-cells, CD45^+^/B220^+^ B-cells, CD45^+^/NK1.1^+^ NK-cells, or CD45^+^/CD11c^+^ dendritic cells in GF/IL17 mice compared with WT mice.

**Table 3:** FACS quantification of infiltrating leukocytes in GF/IL 17 mice and controls relative to all CD45 positive cells.

	Granulocytes	T-cells	B-cells	Nk-cells	Dendritic cells
	(CD45^+^/Ly6G^+^)	(CD45^+^/CD3^+^)	(CD45^+^/B220^+^)	(CD45^+^/Nk1.1^+^)	(CD45^+^/CD11c^+^)
WT	0.3 % ± 0.05	0.5 % ± 0.05	2 % ± 0.7	0.04 % ± 0.01	0.4 % ± 0.07
GF/IL17	0.1 % ± 0.02	0.5 % ± 0.2	1.6 % ± 0.2	0.1 % ± 0.03	0.3 % ± 0.03

### Chronic CNS IL-17A production induces astrocytosis and microglial activation

**Figure 2:**
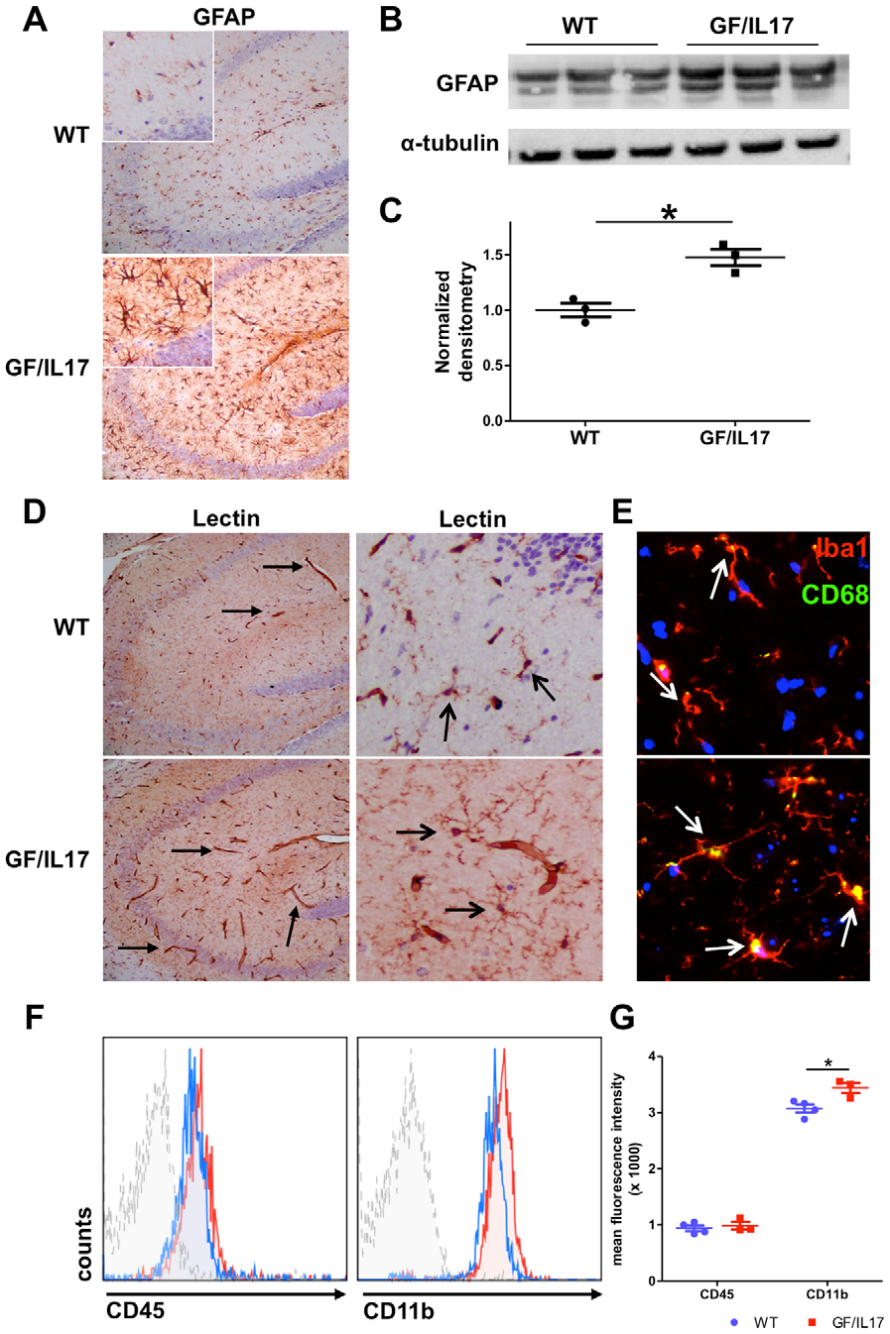
Transgenic CNS expression of IL-17A induces glial activation . **(A)** IHC for GFAP in the hippocampus of WT and GF/IL17 transgenic mice at 9 month. GFAP-staining revealed a strong astrocytic activation by morphological criteria in GF/IL17 mice. **(B)** Astrocytosis was confirmed by anti-GFAP immunoblotting. Whole brain lysates were analyzed by immunoblotting for the presence of GFAP. Anti-tubulin immunoblotting served as internal loading control on the same membrane. **(C)** Densitometric quantifications (arbitrary densitometry units) from immunoblots of B after normalization by tubulin densitometry units obtained from the same immunoblot. (*p < 0.05). **(D)** Tomato-lectin-staining in the hippocampus revealed an activated microglial morphology in GF/IL17A transgenic animals characterized by rounded cell bodies and microglial clustering (open arrows). In addition Lectin staining displayed prominent microvasculature in GF/IL17 mice compared with WT controls (closed arrows; see also Figure 3 for vascular pathology). **(E)** IHC of frozen brain sections for Iba1 (red), CD68 (green) and Dapi (blue). GF/IL17 mice showed a strong immunoreactivity for the activation marker CD68 in Iba1 stained microglia (white arrows indicating colocalisation of the lysosomal markes CD68 and Iba1, age: 9 month). (F) Representative flow cytometric analysis of surface marker expression from freshly isolated microglia in GF/IL17 mice (red) and WT littermate controls (blue). Dashed histogram: isotype control. Histograms were gated on microglial population according to forward/side scatter profile. GF/IL17 mice displayed similar surface expression levels for CD45 compared with WT whereas CD11b expression was upregulated in GF/IL17 mice compared with WT. (G) Statistical analysis of mean fluorescence intensity of freshly isolated microglia in GF/IL17 mice (red) and WT littermate controls (blue). Comparable expression levels of CD45 in GF/IL17 and WT mice whereas CD11b expression levels were significantly upregulated in GF/IL17 mice compared with WT controls (*p < 0.05).

To further examine the effect of chronic IL-17A production on the glial cell population, we characterized the phenotype of astrocytes and microglia by immunohistochemistry. We found evidence for substantial astrocyte activation in GF/IL17 transgenic mice, respectively. In all brain areas from GF/IL17 mice, strong GFAP-immunoreactivity was observed and astrocytes had a swollen cell body and hypertrophic processes characteristic for so called “reactive astrocytes” compared with astrocytes from wild-type control animals (Fig. 2A). The induction of astrocytosis by chronic IL-17A stimulation was confirmed by immunoblotting against GFAP. Corresponding to the reactive astrocytosis observed by immunohistochemistry, western-blots of brain homogenates from GF/IL17 mice showed increased GFAP protein levels compared with wild-type control animals (Fig. 2B). Densitometry revealed a significant increase of GFAP in GF/IL17 mice compared with aged-matched non-Tg littermates (WT: 1.00 ± 0.06 arbitrary units versus GF/IL17: 1.48 ± 0.07 arbitrary units, p ≤ 0.01) (Fig. 2C).

Tomato lectin staining was used to analyse the microglial morphology and state of activation. Typical ramified microglial cells were found in wild-type mice, whereas GF/IL17 mice displayed more intensively labeled microglia with a hypertrophic cell body but still displaying mostly ramified processes (Fig. 2D). To further substantiate the microglial changes observed in GF/IL17 mice, we colocalized microglia with the lysosomal activation marker CD68 and found an increased CD68 staining in microglia from GF/IL17 mice compared with controls (Fig. 2E, arrows). FACS surface marker analysis of freshly isolated microglia confirmed the pronounced microglial activation state in GF/IL17 mice revealing an upregulated surface expression of CD11b compared with littermate controls (Fig. 2F). Statistical analysis of the mean fluorescence intensity of isolated microglia showed only minor effects of IL-17A production on CD45 surface marker expression (MFI - WT: 940.3 ± 59.9 versus GF/IL17: 987.7 ± 67.8, p: n.s.) whereas CD11b surface marker expression was significantly increased by IL-17A stimulation (MFI – WT: 3075 ± 72.1 versus GF/IL17: 3440 ± 89.6, p ≤ 0.05) (Fig. 2G). In summary chronic IL-17 production induces a substantial astrocytosis and microglial activation.

### Chronic CNS IL-17A production induces a vascular pathology with calcification, capillary rarefaction, and thickening of basement membrane but without disruption of the blood brain barrier

**Figure 3:**
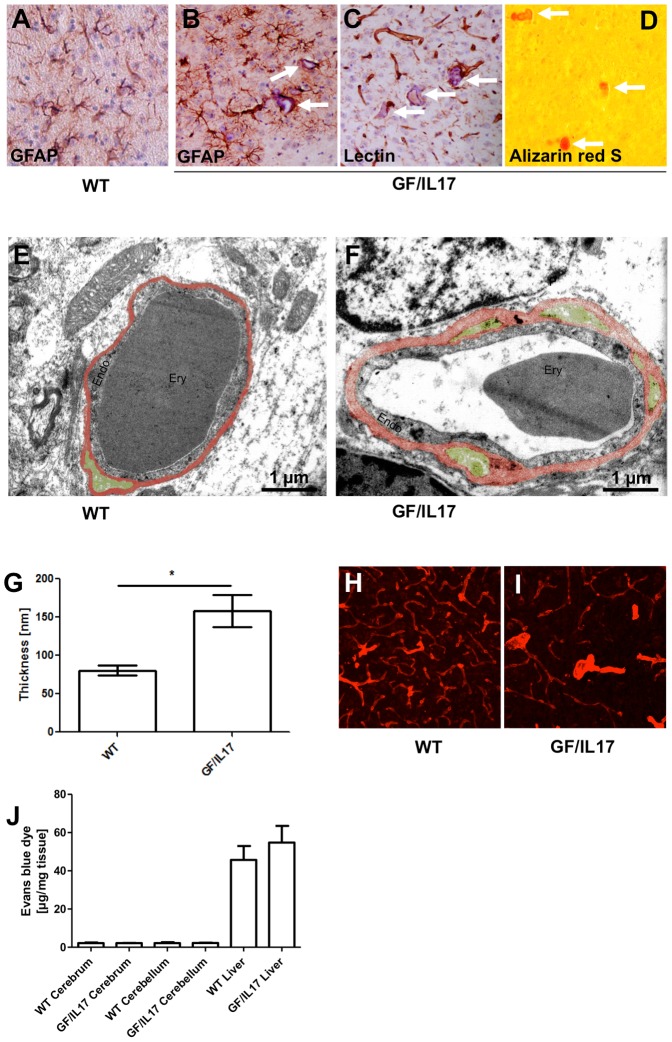
Astrocytic expression of IL-17A induces a vascular pathology with capillary calcifications, microvascular rarefaction and thickening of endothelial layer and basement membrane of vessel walls without disturbing blood-brain barrier integrity. GFAP immunohistochemistry (**A–B**), lectin (**C**) and Alizarin red S staining (**D**) of the thalamus in WT mice (**A**) and GF/IL17 transgenic animals (**C–D**) at the age of 09 month. Microvessels surrounded by GFAP positive astrocytic endfeet and laminin stained endothelia are filled with hematoxillin positive material (white arrows). Around calcified microvessels astrocytes display an activated morphology. Deposits are labelled orange/red in Alizarin red S staining (**D**) confirming vascular calcifications. Transmission electron microscopy of capillaries in the corpus callosum of (**E**) wild-type mice and (**F**) GF/IL17 transgenic mice at the age between 10 and 12 month exhibit morphological criteria of microangiopathy: the endothelial cell layer *(Endo)* appears enlarged compared with WT. Basement membrane *(red-colorored)* surrounding the vascular endothelium appears heavily thickened in TG animals. GF/IL17 mice display numerous duplications of the basement membrane spanning the perivascular space harbouring pericytes *(green colored)*. Inside capillaries erythrocytes *(Ery)* are detectable. Scale bar represents 1 µm. (**G**) Measurement of basement membrane thickness revealed a significant diameter increase in GF/IL17 mice (p < 0,05) (**H**) Confocal microscopy of 50 µm sections labelled with anti-laminin displayed a dense microvascular network in the white matter of WT animals. (**I**) Rarefaction of microvasculature in corresponding areas in GF/IL17 mice. Furthermore arterioles appear thickened. (Age: 10–12 month of both transgenic and wild-type mice) (**J**) To examine blood-brain barrier integrity Evans blue dye (EBD) extravasation into tissue was quantified. Levels of tissue EBD in brains and spinal cord are equal in WT and GF/IL17 mice. Liver tissue served as positive controls.

In addition to the detection of microglial cells, the staining of microglia with tomato-lectin allows the examination of capillaries. Tomato-lectin staining revealed a stronger staining and a different staining pattern with strikingly more prominent vessels in GF/IL17 brain tissue compared to controls (Fig. 2D). Previous studies demonstrated that IL-17A stimulation induces the disruption of blood brain barrier integrity in cerebral microvascular endothelium [39], [46]. We therefore further analyzed the cerebral microvasculature and found numerous small vascular calcifications in aged GF/IL17 transgenic mice located mostly in the thalamic region. No such calcifications were observed in wild-type littermate controls (Fig. 3A) or younger animals of both genotypes suggesting that these bodies appear in an age-dependent manner. Anti-GFAP immunohistochemistry revealed a peri- and intravascular localization of these concrements, which were accompanied by perilesional astrogliosis (Fig. 3B). Lectin histochemistry verified the association of these calcifications to blood vessels (Fig. 3C). The calcium-specific Alizarin Red S staining confirmed that the observed concrements were calcifications (Fig. 3D).

Ultrastructural studies of capillaries within the corpus callosum revealed a marked thickening of the basement membrane in GF/IL17 mice compared with wild-type littermate controls, respectively. Moreover, basement membrane in GF/IL17 mice exhibited profuse duplications marking the perivascular space filled with pericyte processes (Fig. 3E and 3F). Statistical analysis of basement membrane thickness confirmed the significant enlargement of this layer in transgenic mice (WT: 80.7 nm ± 6.7 versus GF/IL17: 158.3 nm ± 21.3, p ≤ 0,05) (Fig. 3G).

To further examine the impact of chronic IL-17A stimulation on the microvascular network, we examined anti-laminin immunofluorescence staining specific for the basement membrane surrounding blood vessels in 50 µm thick brain sections. Predominately in the white matter of forebrain and cerebellum capillary density was reduced in GF/IL17 mice in comparison with controls (Fig. 3H and 3I). To assess the permeability of the blood brain barrier in GF/IL17 transgenic mice, we injected Evańs blue dye (EBD) and determined the release of albumin bound EBD into the brain and spinal cord. None of the brains from either GF/IL17 mice or wild-type controls were macroscopically stained blue whereas tissue distribution of EBD was confirmed by a blue stained liver. Quantifying extravasated EBD from brain lysates further excluded a significant impairment of blood brain barrier function in GF/IL17 mice (Fig. 3J). Again equal tissue distribution of EBD was confirmed by quantifying extravasated EBD from liver lysates of corresponding mice. Additionally we performed an anti-mouse immunoglobulin immunohistochemistry excluding the extravasation of mouse immunoglobulins into the CNS in both GF/IL17 mice and wild-type controls (data not shown).

Taken together these findings indicate that chronic IL-17A production induces a prominent vascular pathology in the CNS with vascular calcification, capillary rarefaction and thickening of the basement membrane. In addition, we could not find evidence for a loss of integrity of the blood-brain barrier in GF/IL17 mice.

### Enhanced upregulation of inflammation related genes and increased numbers of a distinctive population of CD45^high^/CD11b^+^ activated microglia / monocytes in GF/IL17 mice in response to systemic LPS administration

**Figure 4:**
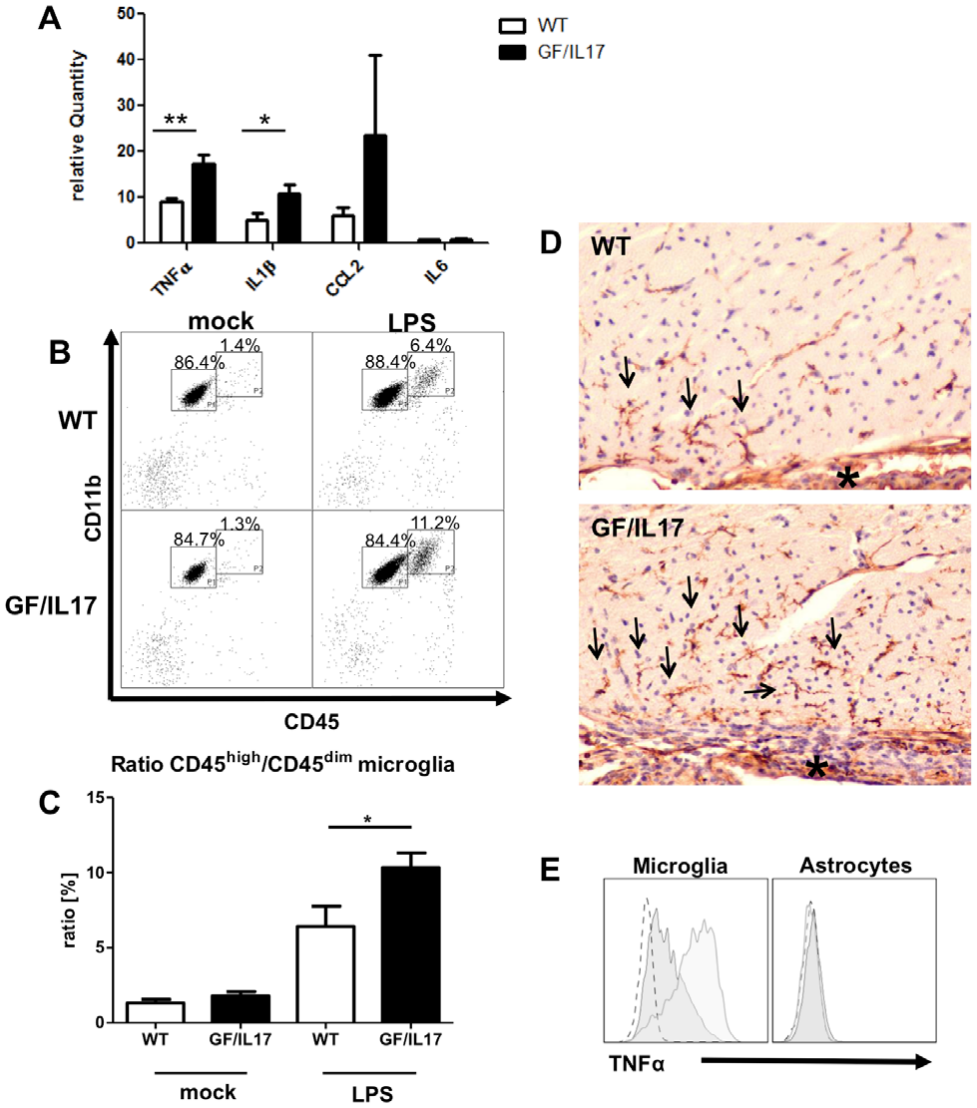
Transgenic IL-17A acts synergistically with other inflammatory stimuli and potentiates LPS induced microgial activation. GF/IL17 transgenic animals and littermate controls between 2 and 3 month were injected twice with 50 µg LPS i.p. in 24 hours or treated with mock injections of PBS. (**A**) Quantitative rt-PCR revealed a strong upregulation of the expression of inflammatory cytokines *Tnf*, *Il1b*, and *Ccl2* by LPS treatment whereas *Il6* was not induced following endotoxemia. This effect was markedly pronounced in GF/IL17 transgenic animals (*Tnf*: p < 0,01; *Il1b*: p < 0,05). Furthermore *Ccl2* expression was strikingly upregulated in some of the LPS treated GF/IL17 mice compared with LPS treated wild-type controls but due to the high interindividual variance not considered as significant. (**B**) Representative flow cytometry profiles from mock- or LPS-treated GF/IL17 and WT mice. LPS treatment induced a population of CD45^high^/CD11b^+^ activated microglia in both WT and GF/IL17 mice. Chronic IL-17A stimulation strikingly augmented this effect, respectively. The numbers above the indicated gate show the mean percentages of FSC/SSC gated populations. (**C**) For the statistical analysis of infiltrating cell numbers a ratio between CD45^high^/CD11b^+^ activated microglia and CD45^dim^/CD11b^+^ resting microglia was calculated for each individual mouse.GF/IL17 transgenic animals exhibited a significantly elevated ratio of CD45^high^/CD11b^+^ activated to CD45^dim^/CD11b^+^ microglia after LPS treatment, respectively (p < 0,05). (**D**) Lectin immunohistochemistry revealed a pronounced accumulation of activated microglia (arrows) in the periventricular regions (asterisk: choroid plexus) after LPS treatment in GF/IL17 mice. (**E**) Intracellular staining of TNF-α after LPS treatment. Only CD45 positive microglia and monocytes/macrophages are stained by anti TNF-α antibody (left histogram) whereas GLAST positive astrocytes are negative for TNF-α (right histogram). In GF/IL17 transgenic mice (light gray) compared with wildtype mice (dark gray) CD45 positive microglia and monocytes/macrophages exhibit a stronger intracellular TNF-α staining after LPS treatment.

The lack of spontaneous tissue destruction and immune cell recruitment into the brain of GF/IL17 mice led us to question if the central production of IL-17 would enhance the local effects of a systemic proinflammatory stimulus. To address this question, we subjected GF/IL17 mice and wild-type littermates to a peripheral immune challenge by intraperitoneal injections of LPS, a model inducing a neuroinflammatory response [69]–[71]. As evidence for modulatory effects of chronic CNS IL-17A production during immune responses, brain homogenates of GF/IL17 mice exhibited a significantly enhanced upregulation of *Tnf* (WT: 9.01 ± 0.74 versus GF/IL17: 17.21 ± 2.02, p ≤ 0.01) and *Il1b* (WT: 5.01 ± 1.41 versus GF/IL17: 10.85 ± 2.03, p ≤ 0.05) mRNA levels after peripheral immune challenge with LPS compared with mock treated wild-type littermates (Fig. 4A). Furthermore mRNA levels of the chemokine *Ccl2* were more pronounced upregulated in GF/IL17 mice compared with LPS treated WT controls, respectively. However, this difference failed to reach statistical significance. Transgenic IL-17A production had no influence on *Il6* mRNA levels in LPS-induced upregulation of inflammatory cytokines in the CNS.

Additional phenotypic analysis of the immune cells in the brain of LPS challenged mice revealed an increase in the numbers of a distinctive CD45^high^/CD11b^+^ population, indicative for activated microglia or accumulating monocytes/macrophages, in GF/IL17 mice compared with wild-type littermates (Fig. 4B). Numbers of CD45^+^/Ly6G^+^ granulocytes, CD45^+^/CD3^+^ T-cells, CD45^+^/B220^+^ B-cells, CD45^+^/NK1.1^+^ NK-cells, or CD45^+^/CD11c^+^ dendritic cells showed no difference, respectively. Statistical analysis of the ratio of the distinctive CD45^high^/CD11b^+^ immune cell population to CD45^dim^/CD11b^+^ resting microglia indicated a significant increase of activated microglia / infiltrating monocytes in GF/IL17 transgenic mice compared with non-transgenic mice (WT: 6.42 % ± 1.36 % versus GF/IL17: 10.33 % ± 0.99 %, p ≤ 0.05) (Fig. 4C).

The characterization of microglial activation by morphological criteria after LPS-induced endotoxemia revealed more activated microglia in GF/IL17 mice compared with wildtype controls (Fig. 4D), which was mostly pronounced in the periventricular region.

To elucidate the cellular source of the increased TNF-α secretion in GF/IL17 mice after LPS-induced inflammation, we performed intracellular cytokine staining and flow cytofluorometric analysis. TNF-α could be detected in CD45 positive microglia/leukocytes but not in GLAST positive astrocytes (Fig. 4E). The level of intracellular TNF-α after LPS treatment in CD45^+^ microglia/leukocytes was increased in GF/IL17 mice compared with wild-type controls consistent with the *Tnf* RNA levels *in vivo*.

Taken together these results point to an augmented neuroinflammatory response in GF/IL17 mice following LPS challenge with increased secretion of proinflammatory cytokines, in particular an increased TNF-α production by microglia, and accumulation of a distinctive CD45^high^/CD11b^+^ leukocyte population in the inflamed CNS.

## Discussion

The cytokine IL-17A has been implicated as an important effector cytokine in various CNS autoimmune and infectious diseases as well as in neurodegenerative processes. To further clarify the functional significance of IL-17A in the CNS, we generated a transgenic mouse line with astrocyte-targeted expression of the murine *Il17a* gene. This approach has been widely and successfully used previously to investigate the function of numerous cytokines and chemokines in the CNS (reviewed in [68]). In several of those transgenic models the local cytokine production by astrocytes is sufficient to initiate and maintain glial activation, leukocyte accumulation and severe CNS tissue injury and functional impairment [57], [58], [72], [73]. However, some of these transgenic models displayed only a very mild or even no phenotype [59], [74]. Thus, the widely varying and unique phenotypes associated with the transgene-encoded production of different cytokines in the CNS highlights not only the specificity of this approach but also the highly selective actions evoked by these factors.

We found that, despite clear evidence of the production of IL-17A in the CNS of GF/IL17 mice, these animals did not develop spontaneous leukocyte infiltration or major tissue destruction. The amount of IL-17A in the CNS of GF/IL17 mice is comparable to IL-17A CNS levels in other neuroinflammatory diseases like EAE, viral or protozoal encephalitis though the cellular sources of this cytokine might differ between our transgenic mouse model and the tested disease models. Consistent with the mild histological phenotype, the transgenic animals did not show any behavioral or physical abnormalities. In addition, no significant alterations in inflammation related gene expression were detected in the brain tissue from transgenic GF/IL17 mice compared with wild-type control littermates. Our findings concerning the impact of chronic IL-17A production on the CNS are in sharp contrast to studies in transgenic mice with overexpression of IL-17 in other organs such as skin or lung [32]: here, IL-17A induced a severe pathology with tissue destruction, which was also observed in a transgenic model with ubiquitous IL17-A overexpression [51]. The differences from our findings likely reflect organ specific factors like the blood brain barrier impeding leukocyte infiltration into the CNS or a reduced response of CNS-resident cells to the IL-17 stimulus. In particular, expression levels and distribution of the IL-17 receptors IL-17RA and IL-17RC might play a role. For IL-17RA highest expression levels have been observed in spleen or kidney whereas brain expression levels were weaker than in the lung [75]. Furthermore lung and skin expression levels of IL-17RC exceed CNS expression levels [76]. Interestingly, the highest IL-17RC expression levels have been shown for vascular endothelium in every examined organ. Our finding, that IL-17A mediates only modest CNS effects whereas severe detrimental effects for this cytokine have clearly been demonstrated for several peripheral organs has also been described for other cytokines as well: e.g. CCL21, which mediates chemotaxis of lymphocytes, induces thyroiditis if expressed under the promotor for thyroglobulin [77] whereas the expression of bioactive CCL21 under the GFAP promoter neither induced lymphocytic infiltration nor glial activation [78]. Therefore our findings underline the unique immunological milieu of the brain concerning cytokine actions.

Although chronic IL-17A production in the CNS does not induce major tissue damage, we found pronounced glial activation in otherwise healthy GF/IL17 mice. The lack of CNS tissue damage is in line with the findings of Haak et al., who demonstrated that transgenic production of IL-17A by T-cells during EAE failed to augment neuroinflammation and tissue damage [51]. In contrast to our findings, Haak et al. did not report any differences in glial activation. However, the transgenic production of IL-17A by T-cells as applied by Haak et al. leads only to increased IL-17 levels at the site of inflammation, where glial cells are already highly activated. Therefore our model of widespread IL17 production in the brain is much more suitable to detect differences in glial cell populations. Astrocytes have been shown to constitutively express the IL-17 receptor, allowing a direct effect of IL-17A on astrocytes [38]. A critical effect of IL-17A on astrocytes was demonstrated by a recent study, identifying astrocytes as a major target cell population of IL-17A stimulation during EAE. This effect was mediated via Act1, a key component in IL-17 signalling [53]. In addition to astrocytosis, GF/IL17 mice displayed microglial cell activation. Microglia have also been shown to constitutively express the IL-17 receptor and microglial cells *in vitro* are known to directly respond to IL-17A stimulation by upregulating proinflammatory cytokines and chemokines [27], [38]. However, we could not find any modulation of these inflammation-related genes in the CNS of the GF/IL17 mice possibly arguing for a counter regulation of IL-17A signalling after chronic stimulation or the necessity for a second stimulus, mediating synergistic effects in combination with IL-17A. Synergistic effects of IL-17 are well described for other cytokine systems like the IL-6 signalling cascade [45] or the IFN-γ dependent iNOS induction in astrocytes [79].

Unexpectedly, GF/IL17 mice developed a vascular phenotype with capillary rarefaction and thickening of the basement membrane, resembling pathological hallmarks of microangiopathy [80], [81]. However, ischemic white matter lesions, which are typical for microangiopathy [80], were not observed in our transgenic model. IL-17A plays an important role in chronic and acute vascular inflammation (reviewed in [20]). A possible link between CNS vascular pathology and IL-17A has recently been published demonstrating an association between bleedings from arteriovenous malformations and a polymorphism in the IL-17A gene in human [82]. There are numerous reports associating IL-17A to the development of arteriosclerotic lesions outside the CNS. In human carotid arteriosclerotic plaques IL-17A is present and upregulated in ruptured plaques of symptomatic patients [83]. In apoE-deficient mice, a well-characterized model for arteriosclerosis, inhibition of IL-17A signalling reduced atherosclerotic lesion formation, prevented plaque rupture, and diminished levels of circulating proinflammatory cytokines [84]-[86]. Furthermore aged GF/IL17 mice displayed vascular calcifications predominately in thalamic regions. This pathological finding is strikingly similar to the thalamic calcifications in vitamin D receptor knockout mice [87], which just recently have been shown to exhibit elevated serum IL-17A levels [88], [89]. Interestingly, a transgenic mouse model with astrocyte targeted expression of TGF-β develops a similar phenotype to GF/IL17 mice: in low expressing TGF-β1 mice there is an age dependent development of perivascular astrocytosis [90], [91] and thickening of the endothelial basement membrane [92]. These findings are suggestive of a common mechanism of IL-17 and TGF-β signalling in mediating vascular pathology, especially as it is known that TGF-β is the most prominent upstream regulator of Th17 differentiation [34]-[37]. Surprisingly the BBB in GF/IL17 mice remained intact. Our finding seemingly contradicts a previous report showing that IL-17A induces reactive oxygen species (ROS) production in brain endothelial cultures, thereby downregulating the tight junction molecule occludin [46]. This discrepancy might be explained by a predominately abluminal secretion of IL-17A in brain vessels in GF/IL17A mice.

Systemic endotoxemia induces cerebral inflammation with upregulation of proinflammatory cytokines and the accumulation of a distinct CD45^high^/CD11b^+^ population. Morphologically these cells seem to be activated microglia but the clear discrimination of this CD45^high^/CD11b^+^ population to CD45^dim^/CD11b^+^ microglia could possibly argue for a recruitment of hematogenous monocytes/macrophages into the CNS. Some studies suggest recruitment of neutrophils and monocytes/macrophages into the CNS in a dose and LPS-strain dependent manner [70], [93], [94]. In GF/IL17 mice as well as in littermate controls we could observe upregulation of proinflammatory TNF-α, IL-1β, and MCP-1 mRNA levels in brain lysates. This induction was significantly enhanced for TNF-α and IL-1β in GF/IL17 transgenic mice arguing for a synergistic effect of IL-17 signalling in neuroinflammatory responses. Additive effects of IL-17A and in example TNF-α have already been shown for numerous cell types [95]-[98].

In summary we have developed a novel transgenic model for the chronic, astrocyte targeted secretion of IL-17A. The findings provide evidence for 1) a minor role of IL-17 to directly induce CNS inflammation; 2) a direct CNS effect of IL-17A by activating glial cells and modulating CNS inflammatory responses and 3) a direct induction of a vascular pathology mimicking pathological hallmarks of microangiopathy. In conclusion, we have generated a novel transgenic model for the CNS directed production of IL-17A which provides a valuable tool for ongoing investigations into the CNS pathobiology of this cytokine.
